# Varicocele-Induced Infertility in Animal Models

**DOI:** 10.22074/ijfs.2015.4234

**Published:** 2015-07-27

**Authors:** Mazdak Razi, Hassan Malekinejad

**Affiliations:** 1 Department of Histology, Faculty of Veterinary Medicine, Urmia University, Urmia, Iran; 2 Department of Pharmacology and Toxicology, Faculty of Veterinary Medicine, Urmia University, Urmia, Iran; 3 Department of Pharmacology and Toxicology, Faculty of Pharmacy, Urmia Medical University, Urmia, Iran Abstract

**Keywords:** Infertility, Inflammation, Oxidative Stress, Varicocele, *In Vitro* Fertilization

## Abstract

Varicocele is characterized by abnormal tortuosity and dilation of the veins of the pampiniform plexus within the spermatic cord. Although several reports show the mechanisms by which the varicocele exerts its infertility impact, the exact pathophysiology for varicocele-induced inflammation and its relationship with testicular endocrine disruption remain largely unknown. This review article will update previous findings by discussing the pathophysiology of long term-induced varicocele in rats. Testicular endocrine disruption in experimentally-induced varicocele, new findings related to biochemical alterations in germinal epithelium, and sperm cells apoptosis are highlighted. Recent observations show that varicocele down-regulates first and second maturation divisions, results in Leydig and Sertoli cell inflammation, and increases immune cell infiltration in the testes of the rat as an animal model. Ultimately, previous findings of our laboratory have revealed that varicocele decreased sperm motility, viability and severe DNA damage. Damage in sperm significantly lowers the animal’s fertility potential. Varicocele not only exerts its pathologic impact by lowering the testicular antioxidant capacity but it also down-regulates first and second maturation divisions by exerting biochemical alterations such as reducing the intracytoplasmic carbohydrate ratio in germinal epithelium.

## Introduction

According to clinical reports, varicocele is observed in 10-20% of the male population, 35-40% of males with primary infertility problems, and up to 80% of men with secondary infertility (1, 2). Annually, 20000 to 40000 infertile men undergo surgery for varicocele (3). Despite numerous studies that emphasize the relation between varicocele and infertility, there are many unsolved questions that remain about the pathophysiology of this impairment. Several studies have shown that varicocele actually causes approximately 35-40% of testicular dysfunction such as damaged seminiferous tubules, a remarkable decrease in Leydig cell distribution and severe testosterone decline in humans (4) and animals, which result in abnormal spermatogenesis and tubules with increased cellular apoptosis (5-7). Increased sperm damage occurs approximately twice (70-85%) as much as seen in testicles and presents as significant decreases in sperm count, motility, viability and remarkable elevations in sperm abnormalities (5, 8, 9). One can hypothesis that the varicocele-induced damages are progressive and a simple pathological analysis of the testicles does not clarify the depth of varicocele-dependent derangements. Thus, the present review focuses on the latest finding of various aspects of varicocele in relation to male infertility in humans. These findings will be compared to the results from our laboratory using rat models with induced varicocele in terms of germinal and sperm cell apoptosis, antioxidant status, inflammation, endocrine function, biochemical changes in carbohydrates, and lipid foci accumulation in the germinal epithelium (6-8). Finally, the varicoceleinduced impact on *in vitro* fertilizing potential of rats will be clarified.

### Current understanding about varicocele pathophysiology

Varicocele develops from retrograde blood flow through the internal spermatic and cremasteric veins into the pampiniform plexus. According to previous observations the venous retrograde blood flow is attributed to the absence of and/or incomplete valves (10, 11). In particular, the reversed blood may lead to severe damage to testicles, partly by two mechanisms: significant resistance to blood flow as measured by the resistive index of capsular branches in varicocele patients (8, 12) and increased scrotal temperature which at least can promote heat-dependent apoptosis (13). Other findings have illustrated that following retrograde blood circulation, the multiple pathophysiologic derangements such as damaged endocrine system, biochemical changes and oxidative stress (6, 14, 15) enhance varicocele-induced impairments. In this regard studies on animal models aim to clarify the pathways where varicocele provokes biochemical changes. Left varicocele induction is used in various studies on animal models in order to induce blood flow into pampiniform plexus (3, 5). We have reduced the left renal vein diameter to less than one mm by ligating the junction of the adrenal and spermatic veins. Then, the anastomotic branch between the left testicular vein and the left common iliac vein was ligated (6, 7).

### Apoptosis in spermatogenesis cell lineage

Spermatogenesis is a proliferative process in which millions of spermatozoa are produced daily. Apoptosis occurs in both pathologic and physiologic conditions as a unique pathway in order to control normal spermatozoa development. In physiologic conditions, apoptosis depends on the capacity of Sertoli cells and mainly occurs in order to eliminate defective germ cells. Thus, it can be considered a critical mechanism to estimate infertility in men (16, 17).

There are many pathways that result in apoptosis in the germinal epithelium; these processes seem to be synchronized in three levels - cellular membrane (18), cytoplasmic (19) and nuclear (20). Apoptosis can affect all three classes of cellular lineages, the spermatogonia, spermatocytes and spermatids (16). At the cell membrane level there are specific membrane receptors which mediate death signals of the tumor necrosis factor receptor family, known as the Fas and Fas ligand (17, 21). Apoptotic cells are recognized by Sertoli cells through binding their membrane receptor to phosphatidylserine, which appears on the surface of the apoptotic germ cells. In this situation the death germ cells are then rapidly phagocytized by Sertoli cells (22, 23).

At the cytoplasmic level there are signal transduction pathways that involve cysteine proteases called caspases (23). At the nuclear level there are specific apoptosis regulatory genes such as *P-53* and *Bcl-2* (24-26). The *P-53* tumor suppressor gene responds to DNA damage and temporarily arrests the cell division cycle at the G_1_ phase. As a result, *P-53* provokes the DNA recovery process (17). However, if the DNA damage is irreparable, *P-53* will stimulate cellular apoptosis via the Fas receptor complex (22). As previously mentioned, varicocele promotes its pathological impact via induction of apoptosis through nuclear and cell membrane levels. We have recently found that in long-time varicocele-induced rats the intracytoplasmic carbohydrates and lipids indirectly participate in germinal cell apoptosis (7).

### Biochemical changes in germinal epithelium and relation with apoptosis at the cytoplasmic level

**Fig.1:**
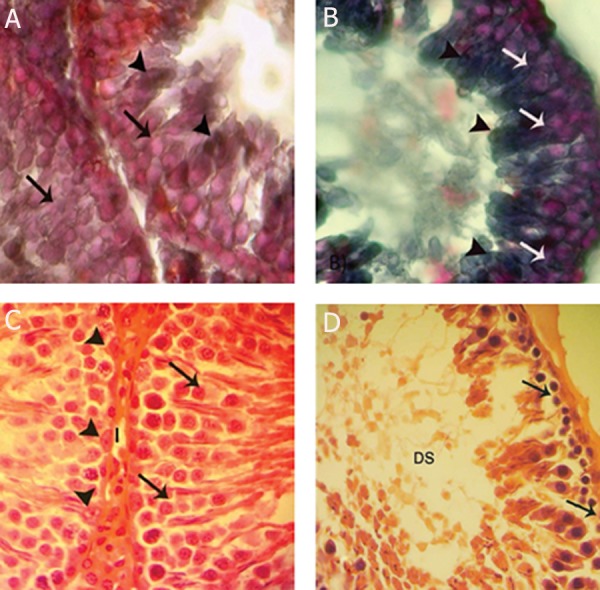
Cross-section from the testes. A. Control group: note spermatogenesis cell lineage with negative Sudan-Black B stained cytoplasm (arrows). The area of spermatogenesis appears with dense reaction sites (head arrows). A comparison of varicocele-induced testis. B. The control group indicates that in non-varicocele-induced testis the spermatogenesis cell line shows faint lipid stained cytoplasm (arrows) and the spermiogenesis area (head arrows) has a dense stained pattern. Varicocele-induced testis is presented with darkly stained cells in all cell lineages (arrows and head arrows). C. Control group: note interstitial connective tissue (I) without edema, Sertoli cells with dense stained cytoplasm (head arrows), spermatogenesis cell lineage with powerful reaction for PAS staining which indicates high cytoplasmic carbohydrate supplement. D. varicocele-induced testis: note faint reaction for PAS. The spermatogonial cells have a negative reaction to PAS staining (head arrows). A, B: Sudan-black B staining; C, D: PAS staining, (x600).

**Fig.2:**
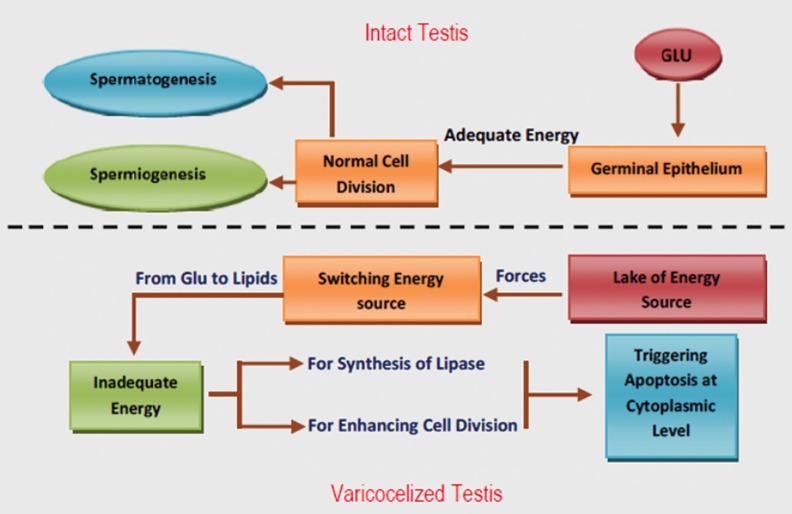
Energy dependent pathways in intact and varicocelized testes; Under normal conditions, glucose (GLU) is transferred the germinal cells via different GLU transporters, which supply enough energy for vital activities of cells such as germinal cell division. In contrast, lack of appropriate sources of energy forces the cells to switch energy sources from GLU to lipids. In order to use lipids the cells need adequate energy to synthesize essential enzymes such as lipase. In varicocele-induced animals synthesis of the lipase enzyme is down-regulated over time. Thus, the cells miss their ability to use the lipids as a secondary source of energy, which leads to remarkable reduction in cell division and continuation of vital functions. The induced impairments trigger cytoplasmic level apoptosis independent of the Fas pathway.

Although there are several reports for nuclear and membrane level apoptosis in varicocele, herein we discuss apoptosis at the cytoplasmic level. In the case that Fas binds to its ligand on Sertoli cells, the generated union forms a complex on the inner surface of the cell which is known as the death-inducing signaling complex that involves procaspase-8. By this mechanism the physiologic elimination of abnormal germinal cells occurs by apoptosis, which is in line with the Sertoli cells physiologic capacity to control normal spermatogenesis (24). Independent to these findings, the glucose transporters I, II, III, IV and VIII mainly mediate transportation of glucose in the spermatogenesis cell lineage (26-28). Since spermatogenesis develops by remarkable utilization of carbohydrates as a main source of energy, any disruption in glucose and/or hexose carbohydrate transport and/or metabolism can promote apoptosis and cellular degeneration in these series of cells (7, 29, 30). Our most recent findings have shown that varicocele-induced rats had significantly decreased carbohydrate content in the spermatogenesis cells versus control animals (7). The cells with faint cytoplasmic carbohydrate content had increased lipid accumulation, at the same time Sertoli cells exhibited a high intracytoplasmic lipid content. In order to explain how varicocele causes enhancement of lipid accumulation in these mentioned cells, one should note, that the lipid supplement in Sertoli cells differs depending on various conditions (Fig.1AD). For instance, when the Sertoli cells begin phagocytosis of residual bodies or damaged cells, the ratio of lipids increases in the cytoplasm of these cells (7, 27, 31, 32). In addition, the varicocele-induced derangements force the cells to switch principal energy from glucose to lipids. The newly selected source of energy will not be able to support cell demands for mitosis (7, 27, 33). Therefore, the cells with defective metabolisms that result from insufficient principal energy sources undergo apoptosis. Ultimately, the involved Sertoli cells begin phagocytosis of the apoptotic cells (Fig.2).

### First and second maturation divisions arrest in varicoceles

There are physiologic correlations (supportive and nutritional) between Sertoli cells and spermatocyte type I cells in the male reproductive system (34, 35). Spermatocyte type I cells are considered to be the precursor cells of spermatogenesis (34- 36). Any detrimental effect on these cells can disrupt the first maturation division which in turn results in severe reduction in the population of cells that undergo second maturation division. Recently we have shown that long-term-induced varicocele in rats resulted in a significant reduction in the maturation division ratio. Accordingly, 58% of the tubules manifested with arrested second maturation division and approximately 40-45% of the tubules were revealed with stopped first maturation division after 8 months. The primary outcome was that the varicocele impacted not only by down-regulation of the first maturation division but it also decreased the second maturation division dependent/ independent to the first division of precursor cells (36).

### Possible mechanisms for maturation division arrest

#### Inflammation

Interleukins (ILs) play an essential role in normal testicular tissue. Physiologic levels of ILs regulate functions of Sertoli and Leydig cells (mainly steroidogenesis) and participate in spermatogenesis (36-38). IL-1β generates severe oxidative stress in different tissues and its compensatory up-regulation in varicoceles testes is well known. Over expression of IL-1β in varicoceles can result in a remarkable increase of reactive oxygen species (ROS) levels which can cause an inflammatory response detrimental to testicular tissue (37). Previously, we have shown that varicocele increased ROS stress in a time-dependent manner. Animals in the eight month–induced varicocele group had remarkable increases in malondialdehyde (MDA) levels accompanied with severely reduced thiol molecule levels in testicles (6). Remarkably increased poly- and mononuclear immune cell infiltration in connective tissue was observed in long-term varicoceles in a rat model. Beside these findings, our observations showed that Sertoli cells in varicocele-induced rats exhibited up-regulated intracytoplasmic alkaline phosphatase levels which suggested that Sertoli cells were directly influenced by inflammation (7, 36). Any inflammatory detrimental effects on Sertoli cells would be able to influence spermatogenesis, particularly at the first maturation division (39, 40). These findings suggested that varicocele-induced inflammation negatively impacted Sertoli cell physiologic function by two mechanisms of extensive ROS stress (via the ILs pathway) and directly by influencing Sertoli cells (alkaline phosphatase positive Sertoli cells). Therefore, damaged Sertoli cells lost their physiologic correlation with the cells that participated in first and second maturation divisions.

### Endocrine system dysfunction

**Fig.3:**
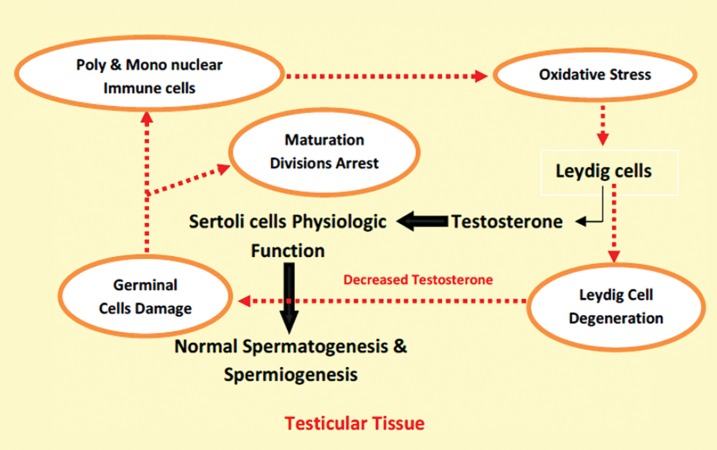
On intact testicular tissue the Leydig cell-produced testosterone promotes Sertoli cell physiologic function, which results in normal spermatogenesis and spermiogenesis processes. On varicocele-exposed testes, the varicocele -induced inflammation impacts the Sertoli cell physiologic role by inhibiting the testosterone synthesis from Leydig cells. The last impairment promotes oxidant generation by two different pathways, including enhancing immune cell infiltration and elevating germinal cell derangement.

A constant level of testosterone at normal concentrations has been clarified as an essential substance to initiate and promote spermatogenesis (41, 42). Serum levels of follicular stimulating hormone (FSH) and luteinizing hormone (LH or ICSH) are extremely important to promote Sertoli and Leydig cell endocrine function. As FSH increases, Sertoli cell synthesis of an androgen binding protein is needed to maintain the high concentration of testosterone (41, 43, 44). LH stimulates the Leydig cells to synthesize essential testosterone. Our experiments on long-term varicoceles in rats have shown a significant reduction in Leydig cell steroidogenesis. Accordingly, the serum level of testosterone remarkably decreased in a time-dependent manner, particularly eight months after varicocele induction, compared to control animals. The histopathological observations for long-term varicoceles testes in rats showed that Leydig cell distribution reduced after eight months. These cells were shown to exhibit vacuolated cytoplasms (7, 36). Although there are contradictory reports about the gonadotropin levels in varicoceles, long-term varicocele-induced rats had decreased serum levels of FSH and LH (7). This disorder might be attributed to the decreased feedback response of Leydig and Sertoli cells to upper axis secreting hormones over time. Thus, it may be concluded that varicocele-induced dysfunction in the endocrine system affects spermatogenesis at both first and second maturation divisions by two mechanisms: a) directly influencing testicular endocrine cells as a-1; reducing Leydig cell distribution and steroid activity accomplished with a-2; and Sertoli cell dysfunction (which it self largely depends on testosterone levels) as well as b) affecting hypophysis-gonadal axis feedbacks (Fig.3).

### Male infertility and oxidative stress

The correlation between oxidative stress and male infertility has been extensively studied (45, 46). ROS include hydrogen peroxide and unstable free radicals with unpaired electrons in their outer orbits. It has been clarified that the mitochondria and plasma membranes of morphologically abnormal and damaged spermatozoa produce ROS through the nicotinamide adenine dinucleotide phosphate-dependent and nicotinamide adenine dinucleotide-dependent oxidoreductase systems, respectively (38). Considering that the duration of the complete spermatogenesis cycle in rats is 45 days (39), normal levels of ROS play an essential role in physiological spermatogenesis, viability, capacitation and sperm motility (38, 47, 48). Excessive ROS generation and/or decreased total antioxidant capacity (TAC) of the testicular tissue result in remarkable increases in ROS levels, which damages the spermatogenesis processes (6, 38, 49). We have shown varicocele reduced testicular antioxidant capacity after eight months in rats by measuring TAC, total thiol molecules (TTM) and MDA levels. The MDA level can be defined broadly as a biomarker for ROS-induced lipid peroxidation. These findings have agreed with previous reports. For example, in some adolescent patients with varicocele, an increased level of MDA indicated extensive lipid peroxidation (50, 51).

### Reactive oxygen species and germinal cell degeneration in varicocele

ROS elevation and/or TAC reduction in the testicular microenvironment of patients with varicocele have been reported (6, 46, 47, 51). The level of 8-Hydroxydeoxyguanosine (8-OHdG), a marker of oxidative stress, and the incidence of 4977bp deletion called "common deletion" (mtDNA^4977^) in mitochondria are increased in varicocele patients. These impairments have been shown to be reversed in patients subjected to varicocelectomy (52, 53). In addition, the end products of lipid peroxidation, such as aldehydes, are alkylating agents that damage cellular DNA and form adducts with proteins that initiate apoptosis (38). Most recently we have shown that the testes samples obtained from six- and eight-month varicocele-induced rats had decreased TAC and TTM levels associated with severe germinal epithelium degeneration (6). A remarkable increase in damaged precursor cells (spermatogonia and spermatocytes), apoptotic spermatozoa and high infiltration of immune cells in testicular tissue, led to a change in the balance between ROS generation and testicular anti-oxidative status. Therefore, animals in the eight-month varicoceles group had the highest level of oxidative stress. Possibly, not only the direct varicoceleinduced damages led to severe ROS generation but also the generated ROS in turn enhanced damage by impairing cells via genomic, mitochondrial and lipid peroxidation-dependent pathways.

### Correlation between increased venous pressure- induced oxidative stress and nitric oxide (NO) in varicocele

Locally produced NO is known to be involved in the regulation of testicular vasculature. In testicular tissue, NO is synthesized from L-arginine by the catalytic activity of two main isoforms of NO synthase-endothelial and inducible NO synthase (NOS). Leydig cells and vasculature of the testes are responsible for the expression of endothelial NOS forms. In varicocele testes, the expression of inducible NOS is enhanced to maintain testicular arterial blood ﬂow as a protective mechanism to elevate blood circulation, which may be detrimental to spermatogenesis. In some adolescent patients with varicocele, increased MDA levels occur together with elevated NO levels, which indicate excessive lipid peroxidation (36). Reaction between produced NO with superoxide anions results in peroxynitrite and peroxynitrous acid generation, which act as powerful oxidants (36, 49, 50). Therefore, it can be concluded that previously produced severe oxidative stress in varicoceles reverses the protective role of NOS into degenerative impact by yielding peroxynitrite and peroxynitrous acid. The generated agents promote oxidative stress-dependent disorders such as DNA damage in varicocelized testes. On the other hand, several studies have shown that NOS plays an important role in provoking heat-dependent apoptosis. Accordingly, the "knock out" of NOS in mice results in improved spermatogenesis, elevated sperm output as well as resistance to heat-induced apoptosis (49, 50). NOS exerts it pathological impact not only by enhancing oxidative stress-dependent disorders, In addition, the produced NOS in varicocele can increase heat-induced apoptosis in testicular tissue.

### Reactive oxygen species and sperm cell physiology in varicocele

**Fig.4:**
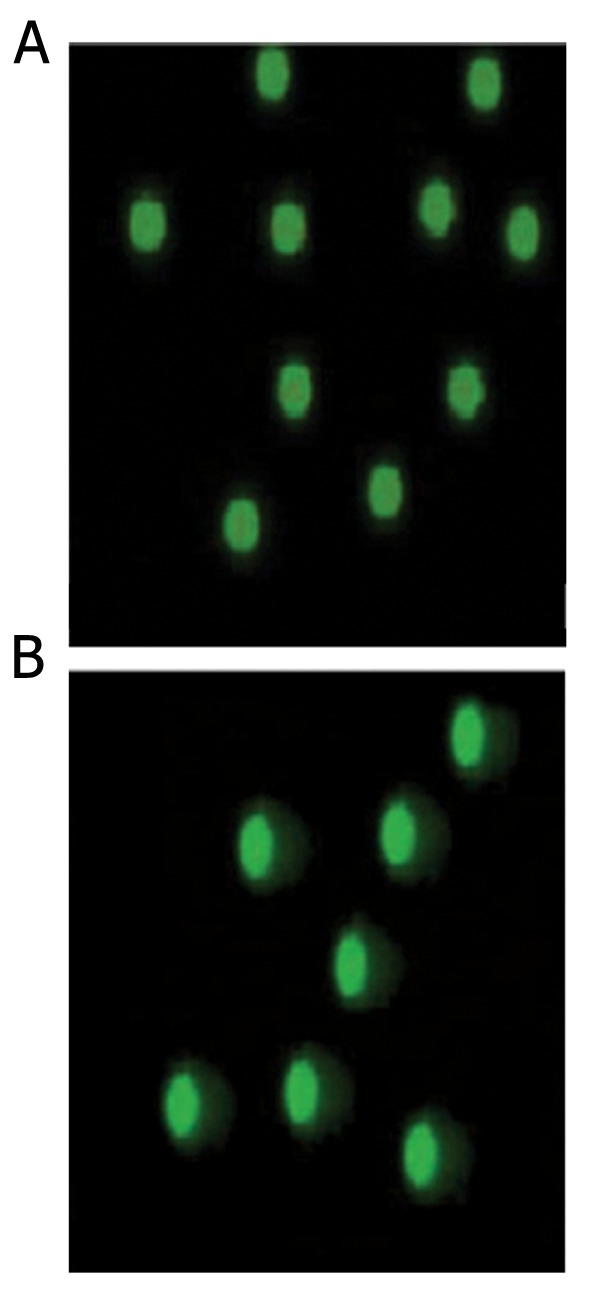
Epi-fluorescent architecture of rat sperm by the Comet assay. A. Sperm from the control group; the green spots without any tails are normal sperm. B. Sperm collected from the left testes of varicocelized rats with intensive DNA fragmentation. The spots with tails indicate DNA fragmentation. Comet assay (x1000).

**Fig.5:**
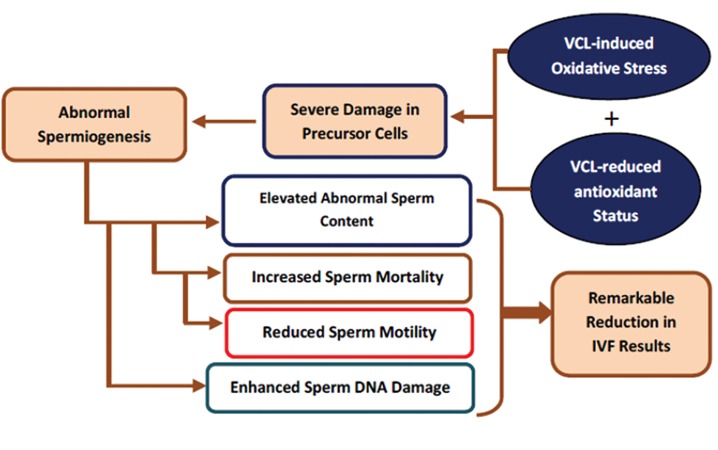
The varicocele (VCL), both by increasing oxidative stress and down-regulating antioxidant capacity, enhance germinal epithelium degeneration which results in abnormal spermiogenesis. Damaged spermiogenesis-induced DNA de-condensation associated with plasma membrane peroxidation via increased reactive oxygen species (ROS) enhances sperm DNA disintegrity and mortality. Oxidative stress-induced damages in sperm axonemal proteins lower sperm motility. Taken together, the sperm fertilizing potential reduces over time, which leads to a low *in vitro* fertilization (IVF) outcome in varicocele patients.

Low amounts of ROS can regulate normal sperm function. Exposure of human spermatozoa against low levels of ROS enhances the sperms’ ability to bind the zona pellucida of the oocyte, whereas the presence of antioxidants reverses this situation (54). Although incubation of sperm with low amounts of oxidants such as H_2_O_2_ can promote sperm capacitation and hyperactivation, the abnormal increased ROS levels pathologically impact sperm. Every ejaculation in humans and even in rodents contains potential sources of ROS, which induce oxidative damage to sperm. The extent of damage largely depends on the amount, duration of exposure, and nature of the oxidants. For example the lipid peroxidation of sperm plasma membrane, immobility and DNA disintegrity enhance depending on the duration of exposure to ROS and extracellular factors such as ions (54, 55). In order to evaluate the effect of long-term induced varicocele on sperm parameters, the epididymis of the animals were dissected out and the caudal section of the epididymal tissue minced in Ham’s F10 culture medium. The sperm were incubated at 37˚C under 5% CO_2_. Vital staining of these sperm showed decreased viability by the time after varicocele induction, which paralleled MDA elevation. The sperm motility and DNA integrity decreased in long-term varicocele-induced rats. Accordingly, the Comet assay for DNA fragmentation showed the highest level of DNA damage in eight-month varicoceles rats (6). Figure 4-A and B show sperm DNA damage. The link between ROS and reduced motility in sperm may be explained by a cascade of events that result in a decrease in axonemal protein phosphorylation as well as oxidant diffusion across the cellular membrane to inhibit the activity of enzymes such as glycerol-3-phosphate dehydrogenase (GPDH). Therefore, the antioxidant defense of sperm decreases, which in turn results in peroxidation of membrane phospholipids (Fig.5) in addition to a severe reduction in sperm motility (6, 55-57).

### Reactive oxygen species-induced damages in sperm and *in vitro* fertilizing ability

Reports indicate that any disorder which leads to a failure in epididymal sperm maturation processes causes disorders to sperm fertilizing ability (58-60). Developments of spermatozoal ability to expose forward motility, such as undergoing capacitation and penetration in the zona pellucida of the oocyte, are examples of several important properties required by the spermatozoa during epididymal sperm passage (61). In order to analyze the effect of long-term induced varicocele on *in vitro* fertilization (IVF) outcome of animals, samples that contained spermatozoa were prepared from sperm suspensions as mentioned earlier. Then, 0.1 ml from superficial sperm of suspensions was added to 150 μl of tissue culture medium (TCM) that contained the oocytes delivered from superovulated normal rats. A drop of medium with 2 oocytes was allocated with a 10 μl sperm suspension (total: 80000 sperm). For each animal, 20 oocytes were divided into 10 drops. Observations showed that the animals in six- and eight-month varicocele-induced groups had the lowest IVF outcomes. In this regard our previous studies showed that the plasma membrane unsaturated fatty acid of sperm have undergone severe damage following varicocele induction (6). These unsaturated fatty acids are essential to give fluidity to the plasma membrane in order to participate in membrane fusion events associated with fertilization. When the associated double bonds with unsaturated fatty acids are deformed, membrane fluidity decreases, leading to a consequent loss of sperm function. Of note, the lowest results for IVF outcome have been shown after eight months. Previous observations showed that embryo development negatively correlated with the level of DNA fragmentation in the germ line (62). Studies showed that DNAdamaged sperms could not fertilize the oocyte (62, 63). According to our findings, sperm from varicoceles rats caused some of the fertilized oocytes to discontinue division in the two-cell embryo phase whereas others were not fertilized at all. Thus, it might be suggested that the loss of fertilizing potential and remarkable reduction in embryonic cell division could be attributed to severe reduction in plasma membrane fluidity as well sperm DNA damage (Fig.5).

## Conclusion

Varicocele associated infertility depends on a cascade of evidence that includes germinal cell apoptosis at the cytoplasmic, nuclear and membrane levels both in human and animal models. Damages that follow experimentally induced varicocele are enhanced. They not only depend on the testicular antioxidant status but also the reduced testicular endocrine function promotes these disorders by stimulating immune cell-dependent inflammation. Finally, development of varicocele-reduced semen quality is time-dependent and negatively impacts sperm fertilizing potential in animal models.
